# Development and validation of the Delirium Burden Scale for Healthcare Providers (DBS‐HCP)

**DOI:** 10.1002/pcn5.70226

**Published:** 2025-10-21

**Authors:** Naoya Ueda, Ichiro Tazaki, Michael LoPresti, Yukiko Shibuya, Shigeru Tokita, Asao Ogawa, Shoki Okuda

**Affiliations:** ^1^ Medical Affairs MSD K.K. Chiyoda‐ku Tokyo Japan; ^2^ INTAGE Healthcare Inc. Chiyoda‐ku Tokyo Japan; ^3^ Exploratory Oncology Research and Clinical Trial Center, National Cancer Center, Division of Psycho‐Oncology Kashiwa Chiba Japan

**Keywords:** burden, delirium, nurse, physician, scale development

## Abstract

**Aim:**

Currently, there is no well‐validated measure intended for use by both physicians and nurses working on general hospital wards that assesses the burden of caring for or treating patients with delirium. To address this unmet need, we aimed to develop and evaluate the reliability and validity of a scale assessing the burden experienced by physicians and nurses treating or caring for patients with delirium on general hospital wards.

**Methods:**

The 25 items for the draft Delirium Burden Scale for HealthCare Providers (DBS‐HCP) were generated from a targeted literature review and qualitative research. Interviews with experts established the scale's content validity. After a pilot survey, 311 physicians and 311 nurses working on general wards in Japan completed the draft DBS‐HCP in the main survey. Statistical analyses evaluated the scale's factor structure, reliability, and validity.

**Results:**

Exploratory factor analysis identified 22 items loading on two factors, which had good reliability (Cronbach's *α* was 0.93 for Factor 1 and 0.89 for Factor 2). In known‐groups validity analysis, burden was higher for nurses than physicians and higher among HCPs who treated or cared for more delirium patients. The correlation between the finalized DBS‐HCP and Japanese Burnout Scale (*r* = 0.34, *p* < 0.001) established construct validity.

**Conclusion:**

We developed DBS‐HCP, a validated scale, to assess the burden of treating or caring for patients with delirium experienced by physicians and nurses working on general wards. Further study is needed to establish the utility of the DBS‐HCP for evaluating interventions to reduce delirium burden.

## INTRODUCTION

Delirium is an acute and common severe neuropsychiatric syndrome characterized by inattention, cognitive impairment, and alteration of consciousness.^1^ The prevalence of delirium varies depending on patient characteristics, clinical settings, and sensitivity of detection methods.^2^, ^3^ Approximately 10%–31% of patients have delirium upon admission to a general hospital, and an additional 3%–29% develop delirium during hospitalization.^2^ Delirium is a well‐known risk factor for cognitive and functional decline, prolonged hospital stays, and increased rates of institutionalization and mortality.^1^

Healthcare providers (HCPs) experience elevated levels of stress, anxiety, and distress when treating or caring for delirious patients.^4^, ^5^, ^6^, ^7^ Distress among physicians and nurses treating or caring for patients with delirium is positively correlated with delirium severity and several specific symptoms associated with delirium, including the presence of hallucinations, delusions, sleep–wake cycle disturbance, and hyperactive delirium‐associated behaviors.^4^, ^5^, ^8^, ^9^, ^10^ Treating or caring for delirium patients is time‐consuming and often associated with anxiety, frustration, helplessness, and safety concerns.^7^, ^9^ Additionally, delirium caused by dehydration and systemic infection is significantly associated with distress in nurses as these etiologies require significant nursing care to treat.^9^ Perceived delirium burden is positively correlated with burnout among physicians and nurses.^4^

Currently available validated tools for assessing the burden of delirium among HCPs are the Strain of Care for Delirium Index (SCDI)^11^ and a scale assessing delirium burden in the intensive care unit (ICU) setting.^12^ The SCDI was designed to measure the difficulty nurses experience when managing patients with delirium and was not intended for or validated for use with physicians. The ICU burden scale is applicable only to nurses working in the ICU setting. A recent publication described the development and psychometric properties of the Delirium‐related Questionnaire (DQ) intended to measure the burden experienced by nurses and physicians in caring for delirious patients.^4^ The main limitations of the DQ are that its items were mainly developed by the author, and a factor analysis was not conducted to confirm the two subscales described by the author.

The purpose of the present study was to develop and validate a new scale, Delirium Burden Scale for Healthcare Providers (DBS‐HCP), to measure the burden experienced by Japanese physicians and nurses when treating or caring for patients with delirium on general hospital wards; such a measure should help document the magnitude of the problem and be useful for evaluating interventions designed to reduce delirium burden experienced by HCPs.

## METHODS

**Figure 1:**
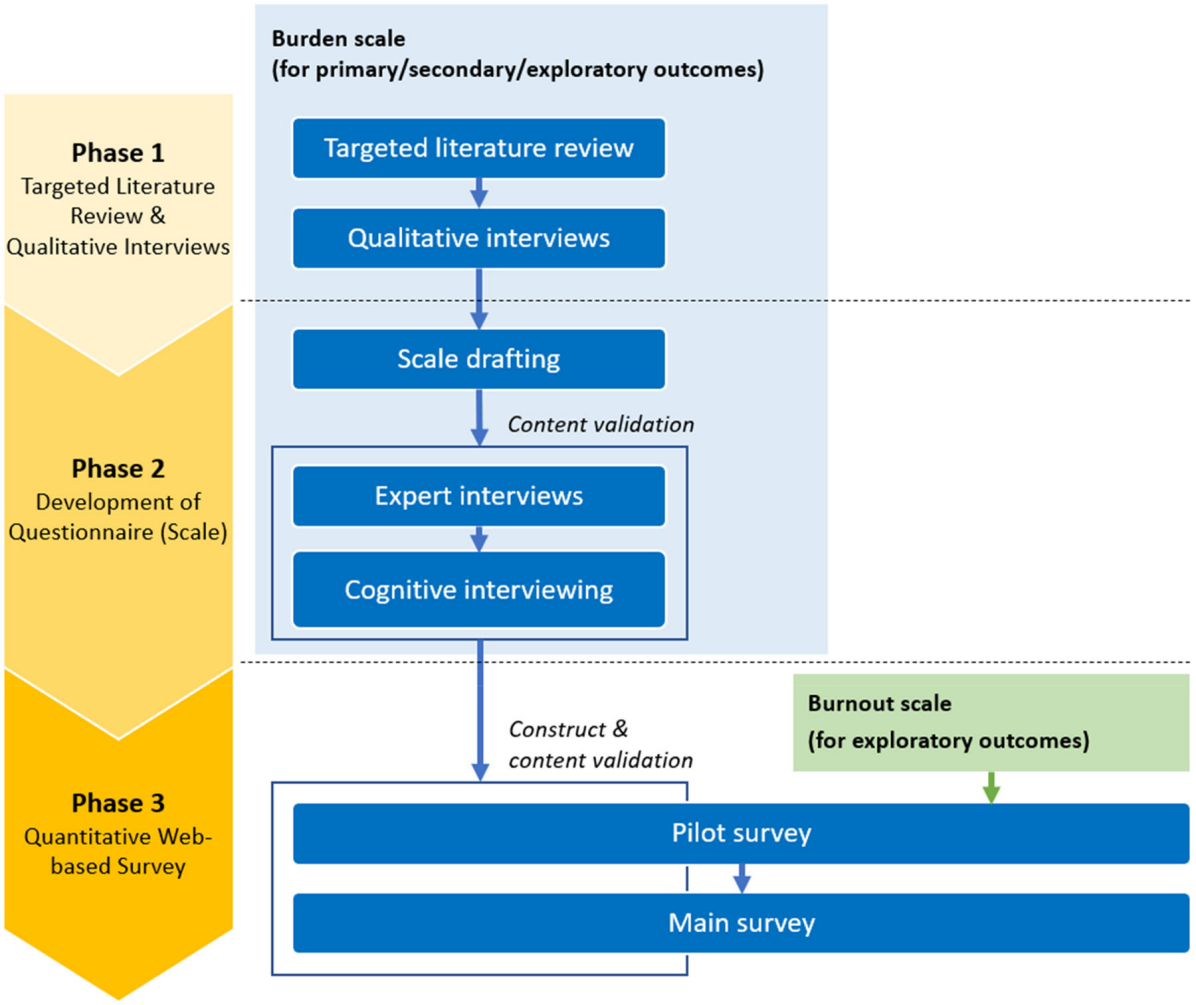
Scale development process.

Figure 1 shows the three‐phase scale development process. In Phase 1, we conducted a targeted literature review and qualitative interviews with HCPs to inform the drafting of the scale items. In Phase 2, we drafted scale items, conducted qualitative interviews with experts to ensure content validity of the draft scale, and used cognitive interviewing with HCPs to assess respondents' understanding of questionnaire items. Phase 3 consisted of a quantitative pilot and main survey. The pilot survey assessed respondents' ease of answering, reliability (internal consistency), and construct validity of the draft scale. The main study assessed the final scale's structure, reliability, and construct validity.

### Targeted literature review and qualitative interviews (Phase 1)

#### Targeted literature review

Three databases were searched—PubMed (Medline), JDream III (JSTPlus and JMEDPlus), and ICHUSHI Web. The search terms and Boolean logic used for each database are shown in Figure S1. Only human studies that were available as full‐text articles written in either English or Japanese were included.

#### Qualitative interviews

Qualitative research involving focus group and one‐on‐one interviews was approved by the Saga Memorial Hospital ethics committee. Eligible qualitative interview participants were physicians and nurses who worked in facilities with at least 20 beds, belonged to an internal medicine, surgical, or emergency department, and cared for patients with delirium at least three times per month. As we aimed to develop a delirium burden scale for use in general hospital settings, individuals working in a psychiatry department or working full‐time in the ICU were excluded.

Qualitative research participants were recruited from the physician and nurse panels managed by PLAMED Inc. A semi‐structured interview guide, informed by the findings of the targeted literature review, was developed to elicit factors related to the burden of care experienced by physicians and nurses. Four 2‐h focus group interviews (two each involving 4 physicians and two each involving 4 nurses) were conducted (16 participants in total). A total of 24 1‐h one‐on‐one in‐depth interviews were conducted with 12 physicians and 12 nurses.

The sample size (20 physicians and 20 nurses) was sufficiently large to achieve concept saturation for each target group. The focus group interviews allowed for a broad elicitation of factors important to physicians and nurses and offered the ability to achieve consensus about items of importance through discussion. The individual interviews allowed for a deeper analysis and probing of factors related to burden and were not subject to bias or self‐imposed constraints due to the presence of and comments made by other participants. The combination of focus group and individual interviews produced a comprehensive elicitation of factors related to delirium burden experienced by physicians and nurses when caring for delirium patients as well as a general prioritization of factors.

Audio recordings of the interviews were transcribed for analysis. Qualitative content analysis was used to identify themes related to burden. A coding scheme was developed involving open and axial coding of the transcripts to extract themes related to burden. Key domains and subdomains relevant to the delirium burden experienced by physicians and nurses were identified. The range and degree of burden for each domain and subdomain were considered. The degree of saturation of the findings was considered based on a review of responses across all the interviews conducted.

Two coders independently coded sequential data transcribed from the qualitative interviews for burden‐related content and categorized content into domains and subdomains identified from the targeted literature review. Any discrepancies between the two coders were resolved by a third coder. All responses for the 40 participants were coded.

### Scale development (Phase 2)

#### Scale drafting

The authors drafted scale items based on findings from the targeted literature review and Phase 1 qualitative research, consistent with recommendations by the International Society for Pharmacoeconomics and Outcomes Research (ISPOR) PRO Good Research Practices Task Force.^13^

#### Expert interviews

To establish content validity and ensure comprehension, the 25‐item draft DBS‐HCP was individually reviewed by three experts in the fields of psychiatry, linguistics, and quality of life/patient‐reported outcome assessment. The three experts were selected and recruited through convenience sampling based on referrals from INTAGE Healthcare Inc. and MSD K.K. Expert interviews, designed to elicit different perspectives on the assessment of delirium burden, were conducted on January 23, 2023, and January 26, 2023. Written and/or typed notes taken during the interviews were compiled, and the draft scale was revised accordingly. No new scale items were generated as a result of the expert interviews.

#### Cognitive interviewing

Content validity and comprehension were further established by conducting cognitive interviews between February 15, 2023, and February 24, 2023, with five physicians and five nurses, including two from internal medicine and one each from surgery, psychiatry, and emergency medicine, who treat and care for patients with delirium. Cognitive interview participants were recruited from the physician and nurse panels managed by PLAMED Inc.; individuals who had participated in Phase 1 qualitative research were excluded from participating in the cognitive interviews. The same inclusion/exclusion criteria used for the Phase 1 qualitative interviews (see above) were applied.

During the cognitive interviews, the items and response options (levels) were evaluated for ease of understanding and response, interpretation, and content validity. Participants were asked about the ease of understanding the instructions, the appropriateness of the recall period, and whether important aspects of burden had been omitted. The cognitive interviews were audio‐recorded and transcribed verbatim. Transcripts were reviewed by the research team to identify aspects of the scale that required improvement to ensure understanding and ease of answering. Modifications to the scale were made based on scores about the ease of understanding and answering, and the appropriateness of items as well as respondents' interpretation of items.

### Quantitative survey (Phase 3)

#### Participants

The pilot and main surveys were approved by the ethics committee at Saga Memorial Hospital. Eligible participants for the surveys were physicians and nurses who worked in facilities with at least 20 beds, belonged to an internal medicine, surgical, or emergency department, and cared for patients with delirium at least one time per month. As we aimed to develop a delirium burden scale for use in general hospital settings, individuals working in a psychiatry department or working full‐time in the ICU were excluded.

#### Measures

The pilot and main surveys were administered online and included separate questionnaires assessing demographics and experience treating delirium patients, the DBS‐HCP, and the Japanese Burnout scale (JBS). The draft DBS‐HCP, administered in the pilot survey and the main survey, consisted of 25 items with a 5‐point Likert scale (1 = disagree strongly, 2 = disagree, 3 = can't say either way, 4 = agree, and 5 = agree strongly); items were equally weighted, and the total score ranged from 25 to 125. The JBS is based on a 17‐item scale originally developed by Tao^14^ and subsequently refined by Kubo,^15^ and consists of three subscales: emotional exhaustion (Domain 1), depersonalization (Domain 2), and personal accomplishment (Domain 3). Participants were asked to rate whether they experienced each of the 17 items on a 5‐point Likert scale from 1 (never) to 5 (always). The JBS uses weighted scoring and has a range of 3 to 15 with higher scores indicating a higher risk of burnout.^16^ In the pilot survey only, one question assessed the comprehension/understanding of each item using a Likert‐type scale (1 = very difficult to understand, 5 = very easy to understand).

#### Statistical analysis

All data analyses were performed using Integrated Development Environment for R (version 4.3, Boston, MA, USA). The pilot survey was conducted to assess the initial reliability (internal consistency) and validity, level of comprehension, and ease of responding to the draft DBS‐HCP. The main survey was conducted with a larger sample to quantitatively examine ceiling and floor effects, evaluate the factor structure of the scale, and establish the reliability (internal consistency) and validity of the finalized DBS‐HCP.

Cronbach's *α* was used to assess the internal consistency of the full draft DBS‐HCP and each factor identified through exploratory factor analysis (EFA) of the final DBS‐HCP. The Kaiser–Meyer–Olkin (KMO), a measure of sampling adequacy, was calculated to examine the adequacy of the factor analysis based on the main survey sample. A high statistical value (0.5–1) indicates that factor analysis is appropriate, while a low statistical value (≤0.5) indicates that factor analysis may be inappropriate. Bartlett's test of sphericity was used to test the hypothesis that the variables included in the survey are uncorrelated. An identity matrix (or population correlation matrix) was used to account for the correlation of each variable. A value *p* < 0.05 indicates that the matrix is not a proper identity matrix and the variables are sufficiently related to each other to perform a meaningful EFA. We calculated the variance proportions, eigenvalues, factor loadings, and factor matrices for the scale items. The factor loadings were examined by the research team based on findings of a previous study.^12^ Items with a factor loading of less than 0.4 on any factor, and items with factor loadings greater than or equal to 0.4 on more than one factor, were excluded.

Ceiling and floor effects were examined for the final DBS‐HCP. The ceiling effect was based on the proportion of responses to DBS‐HCP that were “agree strongly,” and the floor effect by the proportion of responses that were “disagree strongly.” A percentage of 80% or higher for either the ceiling or floor effect may indicate that DBS‐HCP should be modified. We compared the response rate (percentage of respondents who answered “strongly agree” or “agree” to each item) between physicians and nurses using Fisher's Exact test; *p* values were adjusted for multiple comparisons using the Benjamini–Hochberg method.

A known‐groups validity analysis compared scores on the final DBS‐HCP between subgroups using the Kruskal–Wallis test; *p* values were adjusted for multiple comparisons using the Benjamini–Hochberg method. A statistically significant difference was defined as *p* < 0.05 for a 95% confidence interval (CI) and *p* < 0.01 for a 99% CI. The subgroups of interest were type of HCP (physician or nurse), size of facility (≤200 beds or >200 beds), HCP clinical experience (≤5 years or >5 years), HCP experience with delirium care (≤5 years or >5 years), number of delirium patients (less than or equal to the median or greater than the median), and education or training in the treatment and care of delirium (received or did not receive). We hypothesized that nurses and HCPs practicing in larger facilities with less overall experience and less experience with delirium care, a higher number of delirium cases, and lack of education or training in the treatment of delirium would have higher DBS‐HCP scores than, respectively, physicians and HCPs practicing in smaller facilities with more experience overall and in treating delirium, a lower number of delirium cases, and having received education or training in the treatment of delirium.

The Pearson correlation coefficient between total scores on the DBS‐HCP and JBS, which has been widely used in research on burnout,^17^, ^18^, ^19^, ^20^, ^21^ was calculated to demonstrate construct validity. To quantify the overall construct validity, the discriminant coefficient was subtracted from the convergence coefficient. In this calculation, a number close to 1 indicates very high construct validity, while a number below 0.5 indicates limited construct validity; negative values suggest that construct validity is very low. In exploratory analyses, we calculated Pearson correlation coefficients between factors of the DBS‐HCP and domains of the JBS using appropriate formulas.

A multiple regression analysis was performed to confirm the association between physicians' and nurses' burden during delirium care and their delirium‐related experiences and work environment. In the multiple regression analyses, DBS‐HCP scores were the dependent variable and 13 characteristics (Table 3) identified from the literature review and qualitative interviews were the independent variables. Continuous variables were transformed into categorical variables using the median as a cutoff.

## RESULTS

### Targeted literature review and qualitative interviews (Phase 1)

#### Targeted literature review

Figure S2 displays the targeted literature review strategy and results. A total of 1532 potentially relevant articles were identified during the database and head searches for title and abstract screening. Of the 1532 screened articles, 1498 were excluded, and 34 underwent a full‐text review. Of the 34 articles that underwent a full‐text review, 16 were selected as relevant and included in the review.

#### Qualitative interviews

Qualitative data analysis identified the three major themes/domains: Burden related to delirium patients, burden caused by workplace relationships, and burden related to hospital setup and facilities. Each subdomain mentioned by at least 20% (8/40) of participants was assessed by an item on the draft scale. Although there were some differences in the frequency with which physicians and nurses mentioned certain subdomains, there was sufficient overlap between the groups such that a single scale should be applicable to both types of providers.

### Quantitative pilot and main surveys (Phase 3)

#### Participant characteristics

Among 250 physicians and 479 nurses who were invited to participate in the pilot survey, 75 (35 physicians, 40 nurses) completed the survey. Pilot survey sample characteristics are shown in Table S1. The flow of participants enrolled in the main survey is shown in Figure S3. Of the 2375 physicians and 3267 nurses invited to participate, 311 physicians and 311 nurses completed the survey, for response rates of 13.1% and 9.5%, respectively. Characteristics of the 622 main survey participants are shown in Table 1.

**Table 1:** Characteristics of participants.

Characteristic	Physicians (*n* = 311)	Nurses (*n* = 311)	Total (*n* = 622)
Sex, *n* (%)			
Male	294 (94.5)	35 (11.3)	329 (52.9)
Female	15 (4.8)	276 (88.7)	291 (46.8)
Unknown	2 (0.6)	0 (0)	2 (0.3)
Age, *n* (%)			
20–29 years	13 (4.2)	35 (11.3)	48 (7.7)
30–39 years	72 (23.2)	88 (28.3)	160 (25.7)
40–49 years	98 (31.5)	121 (38.9)	219 (35.2)
50–59 years	73 (23.5)	59 (19.0)	132 (21.2)
≥60 years	55 (17.7)	8 (2.6)	63 (10.1)
Facility type			
University hospital	78 (25.1)	41 (13.2)	119 (19.1)
National public hospital	80 (25.7)	85 (27.3)	165 (26.5)
General hospital	150 (48.2)	163 (52.4)	313 (50.3)
Other	3 (1.0)	22 (7.1)	25 (4.0)
Main department			
Internal medicine	147 (47.3)	144 (46.3)	291 (46.8)
Surgery	142 (45.7)	144 (46.3)	286 (46.0)
Emergency care	22 (7.1)	23 (7.4)	45 (7.2)
Facility size			
<100 beds	26 (8.4)	37 (11.9)	63 (10.1)
100–199 beds	47 (15.1)	65 (20.9)	112 (18.0)
200–299 beds	45 (14.5)	38 (12.2)	83 (13.3)
300–399 beds	46 (14.8)	43 (13.8)	89 (14.3)
≥400 beds	147 (47.3)	128 (41.2)	275 (44.2)
No. of delirium patients treated in past 3 months			
Mean (SD)	8.5 (12.0)	16.1 (32.7)	12.3 (24.9)
Median (range)	5 (1–100)	6 (1–300)	5 (1–300)
No. of delirium patients treated in past 1 month			
Mean (SD)	3.6 (4.1)	6.2 (11.7)	4.9 (8.8)
Median (range)	2 (1–30)	3 (1–100)	3 (1–100)
Years of work experience			
Mean (SD)	20.2 (9.7)	17.7 (8.8)	19.0 (9.4)
Median (range)	20.5 (3.2–45.8)	17.3 (0.5–41.5)	19.4 (0.5–45.8)
Years of delirium experience			
Mean (SD)	16.0 (8.5)	11.8 (7.6)	13.9 (8.3)
Median (range)	15.5 (0.4–40.6)	10.0 (0.3–34.6)	12.5 (0.3–40.6)

Abbreviation: SD, standard deviation.

#### Pilot survey findings

The highest percentage of respondents who answered “very difficult to understand” to any item on the newly developed scale was 1.3%. For all items, less than 30% of participants responded “difficult to answer” or “very difficult to understand,” suggesting good comprehension of the survey items. There were no missing or incomplete data. Cronbach's *α* for the 25‐item draft DBS‐HCP was *α* = 0.93 suggesting an acceptable degree of internal validity for the scale. The Pearson correlation coefficient between the draft DBS‐HCP and JBS total scores was not statistically significant (*r* = 0.17, *p* = 0.14).

Table S2 shows the ceiling and floor effects for the 25 items on the draft DBS‐HCP. The percentage of participants who responded “agree strongly” or “disagree strongly” for any item did not exceed 45%. No items were particularly biased towards either ceiling or floor responses.

#### EFA and reliability—Main survey

A scree plot indicated that the DBS‐HCP was comprised of two factors (Figure S4). As a result of the EFA, three items with factor loadings of 0.4 or less were excluded, resulting in a final DBS‐HCP of 22 items with a 5‐point Likert scale (1 = disagree strongly, 2 = disagree, 3 = can't say either way, 4 = agree, and 5 = agree strongly); items were equally weighted and summed such that the total score ranged from 5 to 110. Performing EFA on the 22‐item final DBS‐HCP revealed two factors: Factor 1 (12 items) was defined as the patient‐associated burden of delirium, and Factor 2 (10 items) was defined as the institution‐derived burden of delirium (Table 2). The KMO test was 0.94, and Bartlett's test of sphericity was statistically significant (*Χ* ^2^ = 9374, df = 300, *p* < 0.001). Cronbach's *α* was 0.93 for Factor 1 and 0.89 for Factor 2.

**Table 2:** Descriptive statistics^a^ and factor analysis.

Item	Mean (SD)	Factor 1	Factor 2
1. Felt burdened by the large number of delirious patients	3.9 (1.0)	**0.748**	−0.147
2. Felt burdened by the amount of time spent treating or caring for delirious patients in general	3.9 (0.9)	**0.891**	−0.142
3. Felt burdened by having to care for delirious patients with abnormal behaviors such as violence, verbal abuse, and agitation	4.2 (0.9)	**0.844**	−0.094
4. Felt burdened by having to deal with complications (e.g., tube/catheter removal, falls) related to delirious patients	4.2 (0.8)	**0.826**	−0.151
5. Felt burdened by inability to build communication with delirious patients	3.9 (0.9)	**0.657**	0.071
6. Felt burdened by having to keep an eye on delirious patients	4.1 (0.9)	**0.828**	−0.079
7. Felt burdened by having to prevent or deal with the disturbance of delirious patients because they sometimes have disturbed sleep rhythms or day/night reversal	3.9 (0.9)	**0.640**	0.129
8. Felt burdened by the fact that I could not perform other tasks (gathering information on treatment or care, checking patient information, etc.) because I had to treat or care for delirious patients	3.9 (1.0)	**0.701**	0.116
9. Felt burdened by not being able to sufficiently treat or care for other patients because I had to treat or care for delirious patients	3.9 (0.9)	**0.696**	0.100
10. Felt burdened by explaining their condition, treatment, or care to the family members of delirious patients	3.7 (1.0)	Omitted Item	
11. Felt burdened in responding to inquiries and requests from family members of delirious patients	3.5 (1.1)	Omitted Item	
12. Felt burdened by the ineffectiveness of treatment or care for delirious patients	3.8 (0.9)	**0.518**	0.287
13. Felt burdened to ensure the safety of delirious patients	4.0 (0.8)	**0.756**	0.025
14. Felt burdened by lack of understanding of treatment and compliance with treatment or care (e.g., medications, restraints) among delirious patients	3.9 (0.9)	**0.586**	0.148
15. Felt burdened by not being able to provide sufficient treatment or care, due to my lack of knowledge and experience in treating or caring for delirious patients	3.5 (1.0)	0.145	**0.510**
16. Felt burdened by insufficient cooperation from physicians in the treatment of delirious patients	3.4 (1.1)	0.169	**0.574**
17. Felt burdened by insufficient cooperation from nurses in the care of delirious patients	3.0 (1.1)	−0.190	**0.810**
18. Felt burdened by the differences in attitudes and responses to the treatment or care of delirious patients from other physicians and nurses	3.1 (1.1)	−0.164	**0.810**
19. Felt burdened by the insufficient number of nurses involved in the care of delirious patients	4.0 (0.9)	Omitted Item	
20. Felt burdened by insufficient hospital equipment (cameras, sensor mats, etc.) to treat or care for delirious patients	3.6 (1.1)	0.128	**0.568**
21. Felt burdened by insufficient support (e.g., psychiatrist, psychiatric liaison team) for treatment or care of delirium	3.6 (1.1)	0.059	**0.605**
22. Felt burdened by insufficient room placement considerations and transfers for delirious patients	3.6 (1.0)	0.313	**0.518**
23. Felt burdened by the hospital guidelines and assessment tools that are not in line with the realities of treating or caring for delirious patients	3.4 (1.0)	−0.056	**0.798**
24. Felt burdened by insufficient opportunities to receive education and training on the treatment or care of delirious patients	3.4 (1.0)	−0.117	**0.804**
25. Felt burdened by the lack of environment and facilities for delirious patients to visit their families	3.6 (1.0)	0.007	**0.600**

*Note*: The values in bold indicate items that were assigned to each factor according to the results of factor analysis.

Abbreviation: SD, standard deviation.

^a^ Mean is the average score for each item, which ranges from 1 to 5, with 1 indicating “strongly disagree” and 5 “strongly agree.”

#### Response rate—Main survey

Table S3 shows the response rates (percentage responding “strongly agree” or “agree”) for individual items on the finalized DBS‐HCP for the overall sample and separately for physicians and nurses. Among DBS‐HCP items, highest response rates (“strongly agree” or “agree”) were observed for “Felt burdened by having to deal with complications (e.g., tube/catheter removal, falls) related to delirious patients,” “Felt burdened by having to care for delirious patients with abnormal behaviors such as violence, verbal abuse, and agitation,” and “Felt burdened by having to keep an eye on delirious patients.” A significantly higher proportion of nurses than physicians agreed or strongly agreed with 10 items on the DBS‐HCP.

#### Construct validity — Main survey

**Figure 2:**
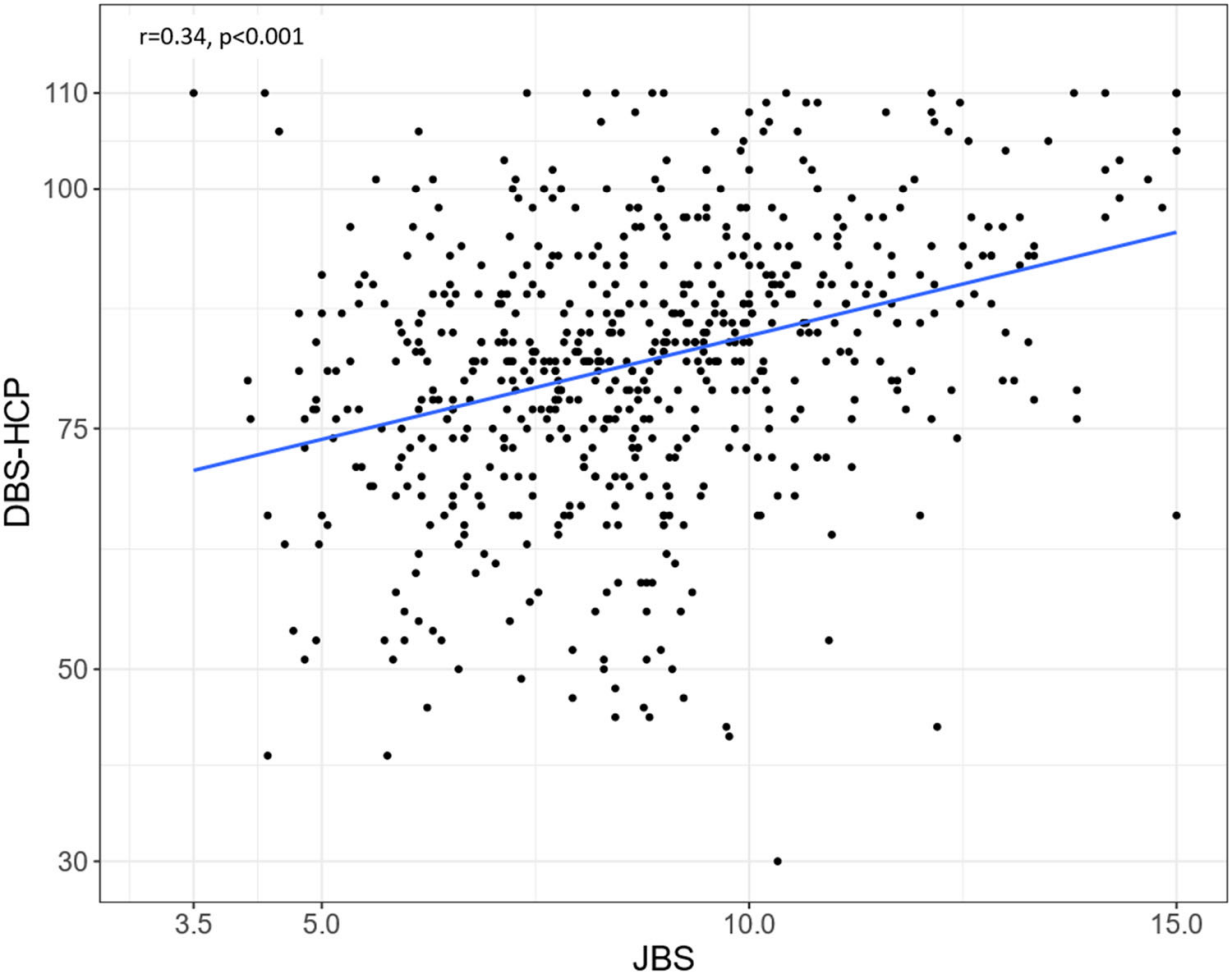
Scatterplot of scores for the Delirium Burden Scale‐Healthcare Provider (DBS‐HCP) and Japanese Burnout Scale (JBS) among all healthcare providers who completed both measures in the main survey (*n* = 622). *r* represents the Pearson correlation between the two scales. The final DBS‐HCP included 22 items scored on a 5‐point Likert scale from 1 = strongly agree to 5 = strongly disagree (range, 22–110). JBS consists of 17 items rated on a 5‐point Likert scale, with the total score calculated using weighted items (range, 3–15).

Figure 2 displays a scatterplot of final DBS‐HCP and JBS total scores (Pearson's *r* = 0.34, *p* < 0.001). Table S4 shows the correlations between factors of the DBS‐HCP and domains of the JBS. DBS‐HCP Factor 1 was most highly correlated with JBS Domain 1 (emotional exhaustion; *r* = 0.38), whereas DBS‐HCP Factor 2 was most highly correlated with both JBS Domain 1 (emotional exhaustion; *r* = 0.28) and JBS Domain 2 (depersonalization; *r* = 0.33).

Table S5 shows the results of the known‐groups validity assessment. Higher total scores on the DBS‐HCP and on both factors were associated with being a nurse and treating or caring for a larger number of patients with delirium.

#### Multiple regression analysis—Main survey

Multiple regression analysis revealed that position (*p* = 0.023), facility type (*p* = 0.022), number of delirium patients treated or cared for in the last month (*p* < 0.001), and frequency of nighttime shifts (*p* = 0.041) were positively correlated with DBS‐HCP total score (Table 3). The results of multiple regression analyses completed separately for physicians and nurses are shown in, respectively, Tables S6 and S7. Among physicians, none of the variables were associated with the DBS‐HCP total score. In contrast, among nurses, the number of delirium patients treated or cared for in the last month and facility type were correlated with the DBS‐HCP total score.

**Table 3:** Multiple regression analysis of variables associated with Delirium Burden Scale for Healthcare Providers (DBS‐HCP).

Variable	*β*	95% confidence interval		*p* value
		Lower	Upper	
Position (nurse)	3.440	0.472	6.407	0.023
Facility type (university hospital)	4.243	0.605	7.880	0.022
Facility size (>200 beds)	−0.115	−3.166	2.936	0.941
Years of experience treating or caring for delirium (>5 years)	−0.011	−0.157	0.135	0.881
Number (higher than median) of delirium patients treated or cared for in the last month	0.235	0.106	0.364	<0.001
Percentage (higher than median) of hyperactive delirium patients treated or cared for in the last month^a^	−0.621	−3.031	1.790	0.613
Experience of education/training in dealing with patients with delirium	0.034	−2.618	2.685	0.980
Existence of delirium clinical path	−1.898	−5.965	2.168	0.359
Existence of management program for patients at risk of delirium	−0.823	−4.204	2.559	0.633
Presence of psychiatrist liaison(s) or interprofessional team collaboration at workplace	−2.126	−5.394	1.142	0.202
Number (higher than median) of physicians in their department	−0.024	−0.248	0.200	0.832
Number (higher than median) of nurses in their department	0.032	−0.059	0.122	0.489
Frequency (higher than median) of nighttime shifts	0.440	0.018	0.863	0.041

*Note*: *β* (partial regression coefficient) is not standardized.

^a^ Percentage (higher than median) of hyperactive delirium patients treated or cared for in the last month. The median percentage of delirium patients who were hyperactive and had been treated or cared for in the past month was 64.6%.

## DISCUSSION

### Psychometric properties of the DBS‐HCP

Using qualitative and quantitative methods, we developed the first well‐validated scale, DBS‐HCP, to assess the burden experienced by physicians and nurses when treating or caring for patients with delirium. The finalized 22‐item DBS‐HCP has no significant ceiling or floor effects and consists of two factors with good internal consistency: patient‐associated burden (Factor 1; Cronbach's *α* 0.93) and institution‐derived burden (Factor 2; Cronbach's *α* 0.89). As expected, higher DBS‐HCP scores were observed in nurses versus physicians and in HCPs who treated or cared for more than the median number of patients with delirium. The DBS‐HCP total score was moderately and significantly correlated with the JBS total score, demonstrating construct validity. In addition, higher delirium burden, whether derived from patients (DBS‐HCP Factor 1) or institutions (DBS‐HCP Factor 2), was significantly associated with greater emotional exhaustion (Domain 1 on the JBS), and higher institution‐derived delirium burden was associated with greater depersonalization (Domain 2 on the JBS). Together, these findings suggest that the burden of delirium has a significant negative impact on the mental health of HCPs as has been shown in previous research.^4^, ^6^, ^7^, ^9^, ^10^, ^22^

### Differences between physicians and nurses

Using the validated DBS‐HCP, the findings of the present study confirm those of a previous study^4^ showing that nurses experience a higher degree of burden of delirium care than physicians. In the previous study, nurses scored significantly higher than physicians on a subset of four items of the 8‐item DQ assessing the perceived frequency and burden of treating or caring for patients with delirium.^4^ In the present study, multivariable regression showed that being a nurse (vs. a physician) was a statistically significant independent predictor of DBS‐HCP total score. Additionally, nurses had significantly higher response rates (answering “agree” or “strongly agree”) than physicians on 7 of 12 items on Factor 1 and on 3 of 10 items on Factor 2. Physicians did not have a higher response rate on any DBS‐HCP item. Together, these findings indicate that nurses experience a higher burden of delirium than physicians who are less involved in hands‐on patient care. Studies of nurses indicate that caring for patients with delirium is very time‐consuming and heavily increases their workload, causing stress, anxiety, and potentially leading to burnout.^5^, ^7^, ^9^, ^10^

### Factors associated with burden

In the multivariable regression, being a nurse, treating or caring for a higher number of delirium patients, working at a university hospital, and working more frequently on night shifts were independent predictors of DBS‐HCP scores. Almost three‐quarters of HCPs (74.1%), with similar rates in physicians and nurses, indicated that they “agreed” or “strongly agreed” that they felt burdened by treating or caring for many delirious patients. It is uncertain why HCPs working in university hospital settings reported greater delirium burden than HCPs working in other hospital settings (e.g., community hospital). We surmise that HCPs working in university hospitals may be more impacted by delirium burden because these settings experience an annual influx of inexperienced healthcare staff during the medical resident turnover period,^23^ have higher patient acuity levels than community hospitals,^24^ and are associated with stressful work.^25^ Shift work, especially night shifts, is associated with higher levels of job strain and other symptoms, such as exhaustion, gastric pain, and lower back pain,^25^ as well as sleep disturbances and their sequelae.^26^ Furthermore, delirium symptoms often worsen at night,^27^ thereby increasing the burden for night shift workers.

### Exposure to education/training about delirium

To help ensure the mental health of HCPs and reduce burnout, hospitals should evaluate the burden of delirium and develop educational/training programs to improve HCPs' knowledge, confidence, and skill in managing delirium. We make this recommendation despite our counterintuitive finding that HCPs who had not been previously exposed to delirium education/training had lower DBS‐HCP Factor 1 scores than HCPs who had received this education/training. This finding may be attributable to selection bias: HCPs with a greater burden of delirium may work in settings with a higher prevalence of delirium, and those settings may be more likely to offer education/training in delirium. Although other studies have shown that receiving education about delirium increases HCPs' knowledge about and confidence in treating or caring for patients with delirium,^28^, ^29^, ^30^, ^31^ it is not clear whether these benefits are retained over time and whether they translate to improved clinical competence and performance in treating and caring for patients with delirium.^32^, ^33^ Given these variable findings, educational approaches for improving management of dementia should be transformed to ensure they have a positive impact on delirium care.^34^

### Most burdensome aspects of treating or caring for patients with delirium

The four DBS‐HCP items with the highest mean responses (most burdensome) and response rate (percentage responding “agree” or “strongly agree”) were dealing with complications related to delirious patients; caring for delirious patients with abnormal behaviors such as violence, verbal abuse, and agitation; having to keep an eye on delirious patients; and ensuring the safety of delirious patients. Similarly, Yamagata et al. found the most burdensome aspects of delirium care for nurses and physicians were patients who removed equipment, such as intravenous (IV) drips and balloons, by themselves; patients who were agitated and violent; restless patients who required so much time and attention that it detracted from their ability to care for other patients; and uncertainty when caring for patients with delirium.^4^

### Limitations and future research

This study had several limitations. First, the response rate to the quantitative survey was low (10%–11%), and physician participants involved in the development of the scale were all male, introducing the possibility of selection bias. Additional research is needed to establish the scale's generalizability. Second, the JBS is an imperfect measure for assessing construct validity because it is a general measure of work‐related stress, whereas the DBS‐HCP is a very specific measure. We were unable to administer the SCDI, delirium burden scale for ICU nurses, or the DQ because no Japanese version of either scale was available at the time the research was conducted. The delirium burden scale for ICU nurses is highly specific to the ICU setting and not applicable to HCPs working on general hospital wards. Third, this study did not find a relationship between years of work experience and DBS‐HCP score, which may be due to highly skewed data (96% of study participants had >5 years of work experience). A previous study found significantly higher delirium burden among ICU nurses with less than 5 years of experience, who accounted for 42% of the sample, than ICU nurses with more experience.^12^ Fourth, although the introduction to the DBS‐HCP acknowledges that there are three subtypes of delirium, the scale does not include items specific to burden associated with hypoactive delirium as, in our qualitative data analyses, burden items specific to hypoactive delirium did not emerge as major sources of burden. Fifth, the sensitivity to change of the DBS‐HCP has not yet been established. If the DBS‐HCP is shown to be sensitive to interventions intended to reduce the burden of delirium experienced by nurses and/or physicians, this would increase the utility of the scale. In recent years, there have been efforts in Japan to develop programs to prevent delirium on general wards, such as the DELirium Team Approach (DELTA) program,^35^, ^36^ but no verified scale has been available to evaluate the effectiveness of these programs for reducing the burden of delirium on medical staff working in general wards. Prospective, longitudinal studies are needed to establish the sensitivity of the DBS‐HCP to change. Sixth, as the primary aim of this study was scale development, additional research is needed to validate the DBS‐CHP scoring method. In addition, research is needed to evaluate the test–retest reliability of the DBS‐HCP and confirm its factor structure. Finally, this study was conducted in Japanese and resulted in the development of a validated Japanese scale (Supporting Information includes the validated final version of the DBS‐HCP). As such, linguistic validation of the instrument is needed before other language versions can be used, including an English version.

### Conclusion

The present study is the first to develop and investigate the reliability and validity of a scale for assessing the burden of treating or caring for patients with delirium on physicians and nurses working on general hospital wards in Japan. The Japanese version of the DBS‐HCP, which consisted of two factors and 22 items, showed good reliability and validity. The DBS‐HCP may be used to quantify the burden of delirium experienced by HCPs in different facilities and across wards in the same facility to determine the need for interventions to reduce burden. Additionally, if future research shows that the scale is sensitive to change, another use of the DBS‐HCP will be to evaluate the effectiveness of burden‐reducing interventions.

## AUTHOR CONTRIBUTIONS

**Naoya Ueda, Ichiro Tazaki, Shigeru Tokita, Asao Ogawa**, and **Shoki Okuda**: Conception, design of the work, interpretation of data, and reviewing this manuscript. **Michael LoPresti** and **Yukiko Shibuya**: Design of the work, acquisition, analysis, interpretation of data, drafting, and reviewing this manuscript. All authors approved the submitted manuscript.

## CONFLICT OF INTEREST STATEMENT

N.U., S.T., I.T., and S.O. are employees of MSD K.K., Tokyo, Japan, a subsidiary of Merck & Co, Inc., Rahway, New Jersey, USA, and may own stock and/or stock options in that company. Michael LoPresti and Yukiko Shibuya are employees of INTAGE Healthcare, which received funding from MSD K.K., Tokyo, Japan, to conduct the study. Asao Ogawa has received funding from MSD K.K., Tokyo, Japan, for research consulting.

## ETHICS APPROVAL STATEMENT

This study was approved by the ethics committee maintained by Saga Memorial Hospital and was conducted according to the principles of the Declaration of Helsinki.

## PATIENT CONSENT STATEMENT

All study participants completed an electronic consent form.

## CLINICAL TRIAL REGISTRATION

Japan Registry of Clinical Trials: jRCT1070220061.

## Supporting information

Supporting Information.

## Data Availability

The data that support the findings of this study are available in the Supporting Information of this article. The dataset generated and analyzed in this survey is not publicly available and cannot be shared with external researchers because participant consent for dataset disclosure was not obtained.
