# Supplementary material for: Development and validation of the Delirium Burden Scale for Healthcare Providers (DBS‐HCP)
Source: PCN Rep. 2025 Oct 21;4(4):e70226. doi: 10.1002/pcn5.70226 (PMC12540204; doi:10.1002/pcn5.70226)

**Supplemental Information**

**Development and validation of the delirium burden scale for healthcare providers (DBS-HCP)**

Naoya Ueda^1^, Ichiro Tazaki^2^, Michael LoPresti^3^, Yukiko Shibuya^4^, Shigeru Tokita^5^, Asao Ogawa^6^, Shoki Okuda^7^

^1^Naoya Ueda

MSD K.K.

Kitanomaru Square

1-13-12, Kudan-kita, Chiyoda-ku

Tokyo, Japan 102-8667

Email: naoya.ueda@msd.com

^2^Ichiro Tazaki

MSD K.K.

Kitanomaru Square

1-13-12, Kudan-kita, Chiyoda-ku

Tokyo, Japan 102-8667

Email: ichiro.tazaki@msd.com

^3^Michael Lopresti

INTAGE Healthcare Inc.

13F Ochanomizu, SolaCity 4-6

Kanda-Surugadai, Chiyoda-ku

Tokyo, Japan 101-0062

Emai: [m.lopresti@intage.com](mailto:m.lopresti@intage.com)

^4^Yukiko Shibuya

INTAGE Healthcare Inc.

13F Ochanomizu, SolaCity 4-6

Kanda-Surugadai, Chiyoda-ku

Tokyo, Japan 101-0062

Email: [yukiko.shibuya@intage.com](mailto:yukiko.shibuya@intage.com)

^5^Shigeru Tokita

MSD K.K.

Kitanomaru Square

1-13-12, Kudan-kita, Chiyoda-ku

Tokyo, Japan 102-8667

Email: shigeru.tokita@msd.com

^6^Asao Ogawa

Psycho-Oncology Division, Exploratory Oncology Research and Clinical Trial Center, National Cancer Centre

6-5-1, Kashiwanoha Kashiwa

Chiba, Japan 277-8577

Email: [asogawa@east.ncc.go.jp](mailto:asogawa@east.ncc.go.jp)

^7^Shoki Okuda (Corresponding Author)

MSD K.K.

Kitanomaru Square

1-13-12, Kudan-kita, Chiyoda-ku

Tokyo, Japan 102-8667

Email: shoki.okuda@msd.com

**Table of Contents**

|  | **Page** |
| --- | --- |
| **Figure S1** | **4** |
| **Figure S2** | **6** |
| **Figure S3** | **7** |
| **Figure S4** | **8** |
| **Table S1** | **9** |
| **Table S2** | **10** |
| **Table S3** | **13** |
| **Table S4** | **15** |
| **Table S5** | **16** |
| **Table S6** | **17** |
| **Table S7** | **18** |
| **Finalized DBS-HCP (Japanese)** | **19** |

**Figure S1. Search terms and Boolean logic by database**

**Database: JDreamⅢ (JSTPlus +JMEDPlus)**

L1　(experience/ALE + 経験/ALE)

L2　(burden/ALE + 負担/ALE)

L3　(lord/ALE + 負荷/ALE)

L4　((adverse effects)/ALE + 悪影響/ALE)

L5　(stress/ALE + ストレス/ALE)

L6　(distress/ALE + 苦痛/ALE)

L7　(Burnout/ALE + 燃え尽き症候群/ALE)

L8　((nurse/ALE + 看護師/ALE) + ナース/ALE)

L9　((doctor/ALE + 医師/ALE) + ドクター/ALE)

L10　(physician/ALE + 内科医/ALE)

L11　(surgeon/ALE + 外科医/ALE)

L12　(psychiatrist/ALE + 精神科医/ALE)

L13　((delirium/ALE + せん妄/ALE) + Delirious/ALE)

L14　(L1 OR L2 OR L3 OR L4 OR L5 OR L6 OR L7) AND (L8 OR L9 OR L10 OR L11 O) L12）

L15　L13 AND L14

L16　L15 AND (JA/LA OR EN/LA) AND (JPN/CY)

L17　L16 AND (Human/ALE + ヒト/ALE)

**Database: ICHUSHI Web**

#1 experience/AL or 経験/AL

#2 burden/AL or 負担/AL

#3 lord/AL or 負荷/AL

#4 adverse/AL and effects/AL or 悪影響/AL

#5 stress/AL or ストレス/AL

#6 distress/AL or 苦痛/AL

#7 Burnout/AL or 燃え尽き症候群/AL

#8 (nurse/AL or 看護師/AL) or ナース/AL

#9 (doctor/AL or 医師/AL) or ドクター/AL

#10 physician/AL or 内科医/AL

#11 surgeon/AL or 外科医/AL

#12 psychiatrist/AL or 精神科医/AL

#13 (delirium/AL or せん妄/AL) or Delirious/AL

#14 #1 or #2 or #3 or #4 or #5 or #6 or #7

#15 #8 or #9 or #10 or #11 or #12

#16 #14 and #15

#17 #13 and #16

#18 (#17) and (LA=日本語,英語)

#19 (#18) and (CK=ヒト)

**Database: PubMed (Medline)**

Search string: ("experience"[Title/Abstract] OR "burden"[Title/Abstract] OR "impact"[Title/Abstract] OR "stress"[Title/Abstract] OR "adverse effects"[Title/Abstract] OR "distress"[Title/Abstract] OR "burnout"[Title/Abstract]) AND ("nurse*"[Title/Abstract] OR "physician*"[Title/Abstract] OR "practitioner*"[Title/Abstract] OR "surgeon*"[Title/Abstract] OR "psychiatrist*"[Title/Abstract]) “ AND ("delirium"[Title/Abstract] OR "delirium"[MeSH Terms])

Filters: Humans, English, MEDLINE

Limits: English language, Humans

**Figure S2. Literature search results**

References from hand searches **(n = 13)**

References identified from databases **(n = 1,530):**

JDreamIII (n = 601)

ICHUSHI Web (n = 402)

PubMed (n = 527)

**\\**

**Identification**

Duplicates removed before screening **(n = 11)**

References excluded **(n = 1,498)**

Title/Abstract screening **(n = 1,532)**

**Screening**

References excluded **(n = 18)**

Did not meet patient criteria (n = 6)

Did not meet outcome criteria (n = 12)

Full-text screening **(n = 34)**

**Included**

References included in review **(n = 16)**

**Figure S3. Participant flow**

**Figure S4. Scree plot for exploratory factor analysis (n=622).** The x-axis refers to the number of principal components or factors. The y-axis refers to the proportion of variance explained by each principal component or factor (eigenvalue). The blue solid line represents factor analysis actual data. The red dotted line represents the factor analysis simulated data.


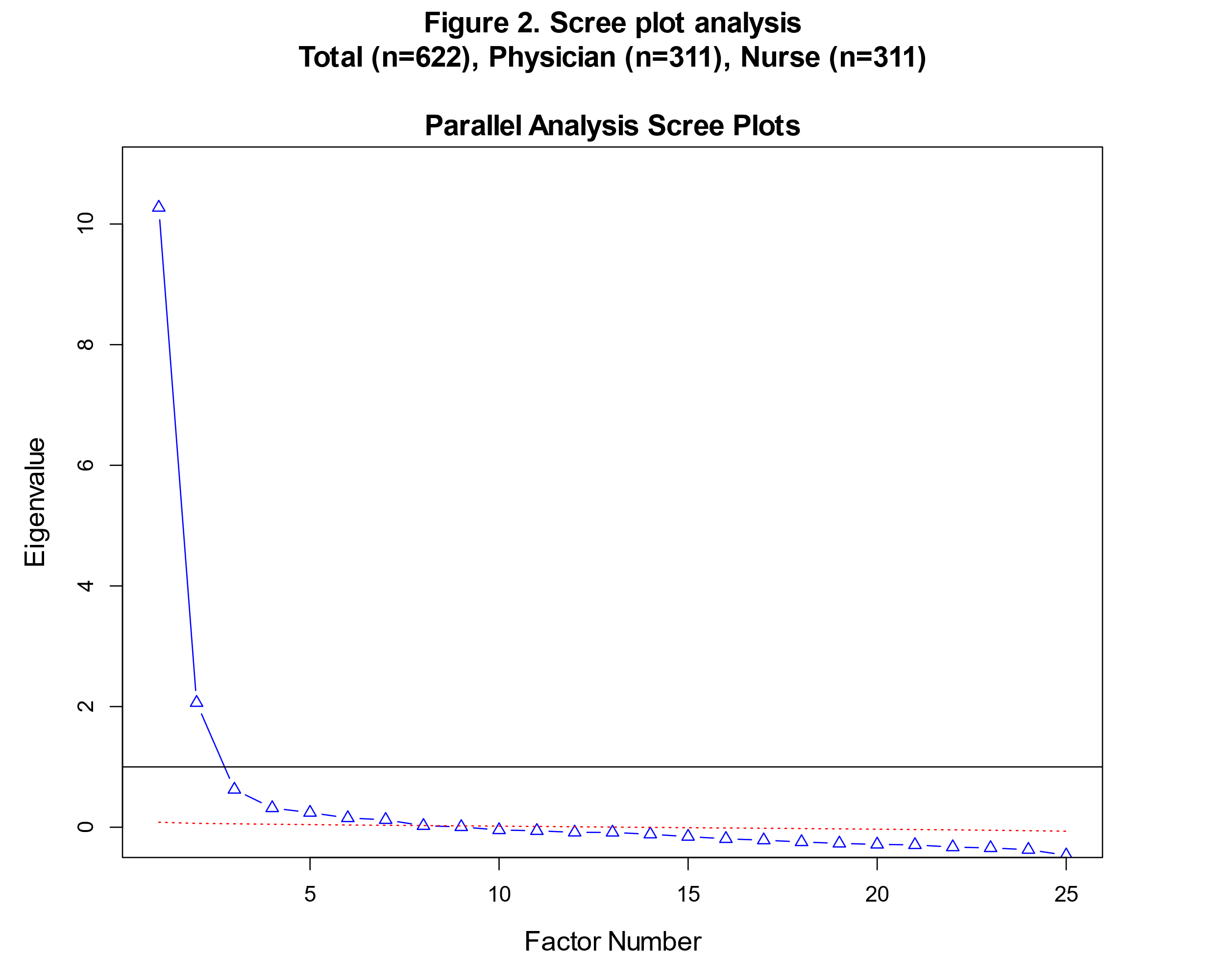
.

**Table S1. Participant characteristics (pilot survey)**

| **Characteristic** | **Physicians**  **(n=35)** | **Nurses**  **(n=40)** | **Total**  **(n=75)** |
| --- | --- | --- | --- |
| Sex, n(%) |  |  |  |
| Male | 30 (85.7) | 5 (12.5) | 35 (46.7) |
| Female | 5 (14.3) | 35 (87.5) | 40 (53.3) |
| Age, n (%) |  |  |  |
| 20-29 years | 1 (2.9) | 3 (7.5) | 4 (5.3) |
| 30-39 years | 8 (22.9) | 10 (25.0) | 18 (24.0) |
| 40-49 years | 9 (25.7) | 13 (32.5) | 22 (29.3) |
| 50-59 years | 8 (22.9) | 13 (32.5) | 21 (28.0) |
| ≥60 years | 9 (25.7) | 1 (2.5) | 10 (13.3) |
| Facility type |  |  |  |
| University hospital | 5 (14.3) | 4 (10.0) | 9 (12.0) |
| National public hospital | 10 (28.6) | 8 (20.0) | 18 (24.0) |
| General hospital | 20 (57.1) | 28 (70.0) | 48 (64.0) |
| Main department |  |  |  |
| Internal medicine | 16 (45.7) | 17 (42.5) | 33 (44.0) |
| Surgery | 16 (45.7) | 17 (42.5) | 33 (44.0) |
| Emergency care | 3 (8.6) | 6 (15.0) | 9 (12.0) |
| Facility size |  |  |  |
| <100 beds | 5 (14.3) | 3 (7.5) | 8 (10.7) |
| 100-199 beds | 5 (14.3) | 5 (12.5) | 10 (13.3) |
| 200-299 beds | 6 (17.1) | 8 (20.0) | 14 (18.7) |
| 300-399 beds | 4 (11.4) | 10 (25.0) | 14 (18.7) |
| ≥400 beds | 15 (42.9) | 14 (35.0) | 29 (38.7) |
| No. of delirium patients treated/cared for in past 3 months |  |  |  |
| Mean (SD) | 9.4 (10.3) | 9.5 (8.9) | 9.5 (9.6) |
| Median (Range) | 6 (1-50) | 8 (1-35) | 6 (1-50) |
| No. of delirium patients treated/cared for in past 1 month |  |  |  |
| Mean (SD) | 4.4 (5.0) | 3.8 (2.9) | 4.1 (4.0) |
| Median (Range) | 3 (1-20) | 3 (1-10) | 3 (1-20) |

SD, standard deviation.

**Table S2. Floor and ceiling effects (main survey)**

| **Item** | **Strongly Agree (%)** | **Strongly Agree or Agree (%)** | **Strongly Disagree (%)** | **Strongly Disagree or Disagree (%)** |
| --- | --- | --- | --- | --- |
| 1. Felt burdened by the large number of delirious patients | 27.3 | 74.1 | 2.6 | 13.3 |
| 1. Felt burdened by the amount of time spent treating or caring for delirious patients in general | 28.0 | 77.2 | 0.6 | 10.0 |
| 1. Felt burdened by having to care for delirious patients with abnormal behaviors such as violence, verbal abuse, and agitation | 40.5 | 85.4 | 0.5 | 6.4 |
| 1. Felt burdened by having to deal with complications (e.g., tube/catheter removal, falls, etc.) related to delirious patients | 42.0 | 87.1 | 0.5 | 4.0 |
| 1. Felt burdened by inability to build communication with delirious patients | 27.5 | 74.3 | 0.3 | 8.4 |
| 1. Felt burdened by having to keep an eye on delirious patients | 40.4 | 81.4 | 0.2 | 7.2 |
| 1. Felt burdened by having to prevent or deal with the disturbance of delirious patients because they sometimes have disturbed sleep rhythms or day/night reversal | 29.1 | 74.0 | 0.3 | 9.6 |
| 1. Felt burdened by the fact that I could not perform other tasks (gathering information on treatment or care, checking patient information, etc.) because I had to treat or care for delirious patients | 31.5 | 72.7 | 0.6 | 11.3 |
| 1. Felt burdened by not being able to sufficiently treat or care for other patients because I had to treat or care for delirious patients | 28.5 | 74.1 | 0.6 | 9.8 |
| 1. Felt burdened by explaining their condition, treatment or care to the family members of delirious patients | 20.4 | 62.5 | 2.3 | 15.4 |
| 1. Felt burdened in responding to inquiries and requests from family members of delirious patients | 18.6 | 55.5 | 2.3 | 19.9 |
| 1. Felt burdened by the ineffectiveness of treatment or care for delirious patients | 20.9 | 72.2 | 0.3 | 8.0 |
| 1. Felt burdened to ensure the safety of delirious patients | 30.4 | 80.2 | 0.2 | 6.8 |
| 1. Felt burdened by lack of understanding of treatment and compliance with treatment or care (e.g., medications, restraints, etc.) among delirious patients | 29.3 | 75.9 | 0.6 | 10.1 |
| 1. Felt burdened by not being able to provide sufficient treatment or care, due to my lack of knowledge and experience in treating or caring for delirious patients | 16.9 | 55.0 | 2.3 | 18.6 |
| 1. Felt burdened by insufficient cooperation from physicians in the treatment of delirious patients | 17.7 | 49.2 | 3.4 | 22.3 |
| 1. Felt burdened by insufficient cooperation from nurses in the care of delirious patients | 10.0 | 34.9 | 7.9 | 38.9 |
| 1. Felt burdened by the differences in attitudes and responses to the treatment or care of delirious patients from other physicians and nurses | 10.3 | 39.1 | 5.9 | 33.3 |
| 1. Felt burdened by the insufficient number of nurses involved in the care of delirious patients | 31.4 | 75.6 | 0.8 | 7.9 |
| 1. Felt burdened by insufficient hospital equipment (cameras, sensor mats, etc.) to treat or care for delirious patients | 20.3 | 57.4 | 2.6 | 18.2 |
| 1. Felt burdened by insufficient support (e.g., psychiatrist, psychiatric liaison team) for treatment or care of delirium | 21.4 | 57.2 | 2.9 | 19.3 |
| 1. Felt burdened by insufficient room placement considerations and transfers for delirious patients | 19.6 | 62.7 | 1.3 | 16.2 |
| 1. Felt burdened by the hospital guidelines and assessment tools that are not in line with the realities of treating or caring for delirious patients | 13.3 | 43.4 | 2.1 | 19.6 |
| 1. Felt burdened by insufficient opportunities to receive education and training on the treatment or care of delirious patients | 12.4 | 46.9 | 1.4 | 21.2 |
| 1. Felt burdened by the lack of environment and facilities for delirious patients　to visit their family | 17.4 | 58.2 | 2.3 | 17.4 |

**Table S3. Number and percentage of participants who responded “strongly agree” or “agree” (response rate) to each item of the final 22-item DBS-HCP overall and by position (main survey)**

| **Item (Factor 1)** | **Overall**  **n (%)** | **Physician**  **n (%)** | **Nurse**  **n (%)** | **P value†** |
| --- | --- | --- | --- | --- |
| 1. Felt burdened by the large number of delirious patients | 461 (74.1) | 221 (71.1) | 240 (77.2) | 0.198 |
| 1. Felt burdened by the amount of time spent treating or caring for delirious patients in general | 480 (77.2) | 216 (69.5) | 264 (84.9) | <0.001 |
| 1. Felt burdened by having to care for delirious patients with abnormal behaviors such as violence, verbal abuse, and agitation | 531 (85.4) | 255 (82.0) | 276 (88.7) | 0.050 |
| 1. Felt burdened by having to deal with complications (e.g., tube/catheter removal, falls, etc.) related to delirious patients | 542 (87.1) | 260 (83.6) | 282 (90.7) | 0.032 |
| 1. Felt burdened by inability to build communication with delirious patients | 462 (74.3) | 230 (74.0) | 232 (74.6) | 0.927 |
| 1. Felt burdened by having to keep an eye on delirious patients | 506 (81.4) | 227 (73.0) | 279 (89.7) | <0.001 |
| 1. Felt burdened by having to prevent or deal with the disturbance of delirious patients because they sometimes have disturbed sleep rhythms or day/night reversal | 460 (74.0) | 222 (71.4) | 238 (76.5) | 0.312 |
| 1. Felt burdened by the fact that I could not perform other tasks (gathering information on treatment or care, checking patient information, etc.) because I had to treat or care for delirious patients | 452 (72.7) | 188 (60.5) | 264 (84.9) | <0.001 |
| 1. Felt burdened by not being able to sufficiently treat or care for other patients because I had to treat or care for delirious patients | 461 (74.1) | 189 (60.8) | 272 (87.5) | <0.001 |
| 1. Felt burdened by the ineffectiveness of treatment or care for delirious patients | 449 (72.2) | 219 (70.4) | 230 (74.0) | 0.482 |
| 1. Felt burdened to ensure the safety of delirious patients | 499 (80.2) | 237 (76.2) | 262 (84.2) | 0.038 |
| 1. Felt burdened by lack of understanding of treatment and compliance with treatment or care (e.g., medications, restraints, etc.) among delirious patients | 472 (75.9) | 230 (74.0) | 242 (77.8) | 0.475 |
| **Item (Factor 2)** |  |  |  |  |
| 1. Felt burdened by not being able to provide sufficient treatment or care, due to my lack of knowledge and experience in treating or caring for delirious patients | 342 (55.0) | 178 (57.2) | 164 (52.7) | 0.475 |
| 1. Felt burdened by insufficient cooperation from physicians in the treatment of delirious patients | 306 (49.2) | 124 (39.9) | 182 (58.5) | <0.001 |
| 1. Felt burdened by insufficient cooperation from nurses in the care of delirious patients | 217 (34.9) | 104 (33.4) | 113 (36.3) | 0.551 |
| 1. Felt burdened by the differences in attitudes and responses to the treatment or care of delirious patients from other physicians and nurses | 243 (39.1) | 116 (37.3) | 127 (40.8) | 0.503 |
| 1. Felt burdened by insufficient hospital equipment (cameras, sensor mats, etc.) to treat or care for delirious patients | 357 (57.4) | 153 (49.2) | 204 (65.6) | <0.001 |
| 1. Felt burdened by insufficient support (e.g., psychiatrist, psychiatric liaison team) for treatment or care of delirium | 356 (57.2) | 172 (55.3) | 184 (59.2) | 0.482 |
| 1. Felt burdened by insufficient room placement considerations and transfers for delirious patients | 390 (62.7) | 154 (49.5) | 236 (75.9) | <0.001 |
| 1. Felt burdened by the hospital guidelines and assessment tools that are not in line with the realities of treating or caring for delirious patients | 270 (43.4) | 130 (41.8) | 140 (45.0) | 0.540 |
| 1. Felt burdened by insufficient opportunities to receive education and training on the treatment or care of delirious patients | 292 (46.9) | 148 (47.6) | 144 (46.3) | 0.848 |
| 1. Felt burdened by the lack of environment and facilities for delirious patients to visit their family | 362 (58.2) | 175 (56.3) | 187 (60.1) | 0.482 |

†P value is between physicians and nurses and was calculated using the Fisher’s Exact test; P values were adjusted for multiple comparisons using the Benjamini-Hochberg method.

**Table S4. Pearson correlation coefficients between factors of delirium burden scale for healthcare providers (DBS-HCP) and domains of the Japanese burnout scale (JBS) — Main survey**

|  | | **DBS-HCP** | | **JBS** | | |
| --- | --- | --- | --- | --- | --- | --- |
|  |  | **Factor 1** | **Factor 2** | **Domain 1** | **Domain 2** | **Domain 3** |
| DBS-HCP | Factor 1 | — | 0.58 | 0.38 | 0.20 | 0.12 |
|  | Factor 2 | 0.58 | — | 0.28 | 0.33 | 0.05 |
| JBS | Domain 1 | 0.38 | 0.28 | — | 0.72 | 0.30 |
|  | Domain 2 | 0.20 | 0.33 | 0.72 | — | 0.21 |
|  | Domain 3 | 0.12 | 0.05 | 0.30 | 0.21 | — |

**Table S5. Known-groups analysis (main survey)**

|  | **Physician (n=311)** | **Nurse (n=311)** | **P value†** |
| --- | --- | --- | --- |
| Total score | 78.6 (13.8) | 85.4 (13.4) | <0.001 |
| Factor 1 | 45.6 (7.9) | 50.2 (7.9) | <0.001 |
| Factor 2 | 33.0 (7.2) | 35.1 (7.6) | 0.002 |
|  | **≤200 beds (n=175)** | **>200 beds (n=447)** |  |
| Total score | 82.2 (13.7) | 81.9 (14.1) | 0.666 |
| Factor 1 | 47.7 (8.5) | 48.0 (8.2) | 0.897 |
| Factor 2 | 34.6 (6.9) | 33.9 (7.7) | 0.398 |
|  | **≤5 years work experience**  **(n=27)** | **>5 years work experience (n=593)** | **P value** |
| Total score | 84.7 (9.8) | 81.9 (14.2) | 0.514 |
| Factor 1 | 50.3 (6.6) | 47.8 (8.3) | 0.362 |
| Factor 2 | 34.4 (7.2) | 34.1 (7.5) | 0.897 |
|  | **≤5 years delirium care/treatment (n=75)** | **>5 years delirium care/treatment (n=542)** | **P value** |
| Total score | 81.8 (13.5) | 82.0 (14.1) | 0.897 |
| Factor 1 | 48.0 (8.3) | 47.9 (8.3) | 0.985 |
| Factor 2 | 33.9 (7.1) | 34.1 (7.6) | 0.819 |
|  | **≤median number of inpatients cared/treated for delirium (n=401)** | **>median number of inpatients cared/treated for delirium (n=221)** | **P value** |
| Total score | 80.9 (13.7) | 84.0 (14.3) | 0.010 |
| Factor 1 | 47.3 (8.0) | 49.0 (8.6) | 0.023 |
| Factor 2 | 33.5 (7.4) | 35.0 (7.6) | 0.033 |
| **Item** | **With education/training on the care/treatment of delirium (n=280)** | **Without education/training on the care/treatment of delirium (n=342)** | **P value** |
| Total score | 82.7 (13.1) | 81.4 (14.7) | 0.405 |
| Factor 1 | 49.0 (7.6) | 47.0 (8.6) | 0.017 |
| Factor 2 | 33.7 (7.6) | 34.4 (7.4) | 0.405 |

All values are mean (standard deviation) unless otherwise specified.

†P values compare physicians and nurses and were calculated using the Kruskal-Wallis test; P values were adjusted for multiple comparisons using the Benjamini-Hochberg method.

**Table S6. Multiple regression analysis of variables associated with DBS-HCP score (physicians) — Main survey**

| **Variable** | **β** | **95% Confidence Interval** | | **P value** |
| --- | --- | --- | --- | --- |
|  |  | **Lower** | **Upper** |  |
| Facility type (university hospital) | 1.113 | -3.731 | 5.957 | 0.651 |
| Facility size (>200 beds) | 1.441 | -3.603 | 6.485 | 0.574 |
| Years of experience treating or caring for delirium （>5 years) | -0.208 | -0.421 | 0.006 | 0.056 |
| Number (higher than median) of delirium patients treated or cared for in the last month | 0.302 | -0.110 | 0.715 | 0.150 |
| Percentage (higher than median) of hyperactive delirium patients treated or cared for in the last month* | -2.412 | -6.050 | 1.225 | 0.192 |
| Experience of education/training in dealing with patients with delirium | -0.675 | -4.559 | 3.209 | 0.732 |
| Existence of delirium clinical path | 1.924 | -4.073 | 7.920 | 0.528 |
| Existence of management program for patients at risk of delirium | -1.027 | -5.927 | 3.874 | 0.680 |
| Presence of psychiatrist liaison(s) or interprofessional team collaboration at workplace | -4.031 | -9.295 | 1.232 | 0.133 |
| Number (higher than median) of physicians in their department | 0.185 | -0.191 | 0.561 | 0.333 |
| Number (higher than median) of nurses in their department | -0.016 | -0.133 | 0.101 | 0.791 |
| Frequency (higher than median) of nighttime shifts | 0.408 | -0.274 | 1.091 | 0.240 |

β (partial regression coefficient) is not standardized.

*Percentage (higher than median) of hyperactive delirium patients treated or cared for in the last month. The median percentage of delirium patients who were hyperactive who had been treated or cared for in the past month was 64.6%.

**Table S7. Multiple regression analysis of variables associated with DBS-HCP score (nurses) — Main survey**

| **Variable** | **β** | **95% Confidence Interval** | | **P value** |
| --- | --- | --- | --- | --- |
|  |  | **Lower** | **Upper** |  |
| Facility type (university hospital) | 6.249 | 0.386 | 12.113 | 0.037* |
| Facility size (>200 beds) | -1.815 | -5.697 | 2.066 | 0.358 |
| Years of experience treating or caring for delirium （>5 years) | 0.166 | -0.040 | 0.373 | 0.114 |
| Number (higher than median) of delirium patients treated or cared for in the last month | 0.221 | 0.086 | 0.356 | 0.001* |
| Percentage (higher than median) of hyperactive delirium patients treated or cared for in the last month* | 1.580 | -1.666 | 4.825 | 0.339 |
| Experience of education/training in dealing with patients with delirium | 1.576 | -2.119 | 5.271 | 0.402 |
| Existence of delirium clinical path | -5.314 | -11.072 | 0.443 | 0.070 |
| Existence of management program for patients at risk of delirium | -1.275 | -6.067 | 3.517 | 0.601 |
| Presence of psychiatrist liaison(s) or interprofessional team collaboration at workplace | -1.679 | -5.892 | 2.533 | 0.433 |
| Number (higher than median) of physicians in their department | -0.209 | -0.491 | 0.073 | 0.146 |
| Number (higher than median) of nurses in their department | 0.139 | -0.019 | 0.297 | 0.084 |
| Frequency (higher than median) of nighttime shifts | 0.461 | -0.076 | 0.998 | 0.092 |

β (partial regression coefficient) is not standardized.

*Percentage (higher than median) of hyperactive delirium patients treated or cared for in the last month. The median percentage of delirium patients who were hyperactive who had been treated or cared for in the past month was 64.6%.

**The original (Japanese) version of the DBS-HCP**

**
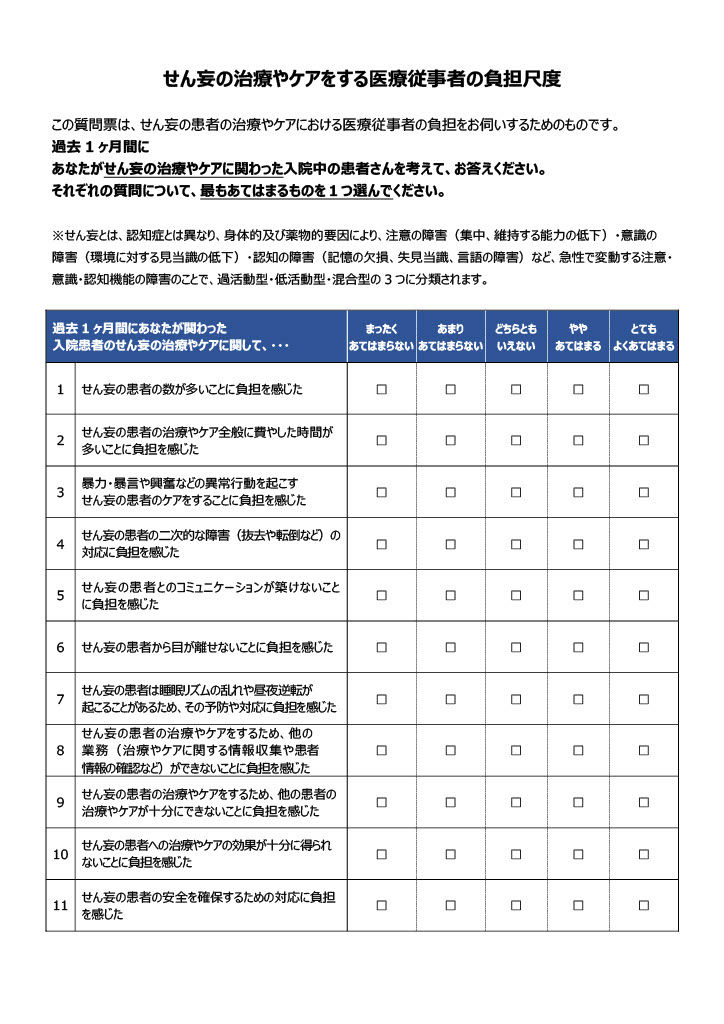
**


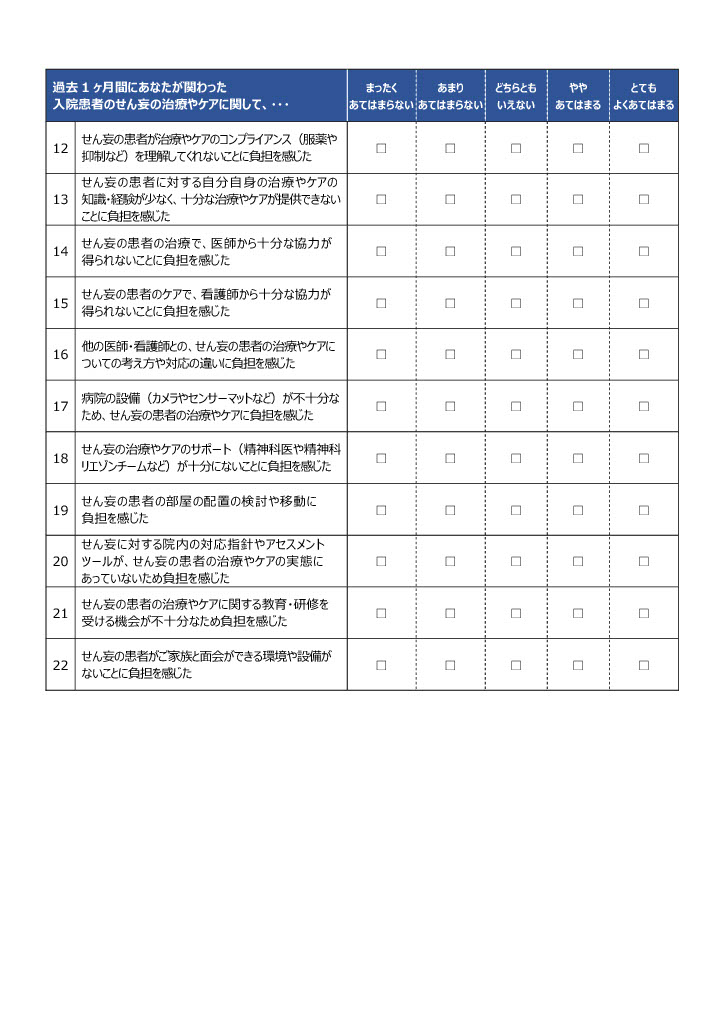

Supplement: Supplementary file 1 — Supporting Information. [file PCN5-4-e70226-s001.docx]
